# Characterization of a Panel of Monoclonal Antibodies Targeting the F-Protein of the Respiratory Syncytial Virus (RSV) for the Typing of Contemporary Circulating Strains

**DOI:** 10.3390/tropicalmed9010001

**Published:** 2023-12-19

**Authors:** Vera Krivitskaya, Ekaterina Petrova, Evgeniy Sorokin, Tatyana Tsareva, Maria Sverlova, Kseniia Komissarova, Anna Sominina, Daria Danilenko

**Affiliations:** Smorodintsev Research Institute of Influenza, The Ministry of Health of the Russian Federation, WHO National Influenza Centre, St. Petersburg 197376, Russia; ekaterina.petrova@influenza.spb.ru (E.P.); evgeniy.sorokin@influenza.spb.ru (E.S.); tatyana.tsareva@influenza.spb.ru (T.T.); maria.sverlova@influenza.spb.ru (M.S.); kseniya.sintsova@influenza.spb.ru (K.K.); anna.sominina@influenza.spb.ru (A.S.); daria.danilenko@influenza.spb.ru (D.D.)

**Keywords:** respiratory syncytial virus, contemporary circulating strains, F protein, monoclonal antibody, escape mutant, fixed cell-ELISA, immunoblotting, neutralization activity

## Abstract

Respiratory syncytial virus (RSV) is the most common cause of upper and lower respiratory tract infections in infants and young children. Virus-specific monoclonal antibodies (mAbs) can be used for diagnosis, prophylaxis, and research of RSV pathogenesis. A panel of 16 anti-RSV mAbs was obtained from mice immunized by RSV strain Long. Half of them had virus-neutralizing activity. According to Western blot all of these mAbs effectively bound native oligomeric (homodimeric and homotrimeric) forms of the RSV fusion (F) protein. Only five of the mAbs interacted with the monomeric form, and only one of these possessed neutralizing activity. None of these mAbs, nor the commercial humanized neutralizing mAb palivizumab, reacted with the denaturated F protein. Thus, interaction of all these mAbs with F protein had clear conformational dependence. Competitive ELISA and neutralization assays allowed the identification of nine antigenic target sites for the interaction of mAb with the F protein. Five partially overlapping sites may represent a complex spatial structure of one antigenic determinant, including one neutralizing and four non-neutralizing epitopes. Four sites (three neutralizing and one non-neutralizing) were found to be distinct. As a result of virus cultivation RSV–A, strain Long, in the presence of a large amount of one of the neutralizing mAbs, an escape mutant with a substitution, N240S, in the F protein, was obtained. Thus, it was shown for the first time that position 240 is critical for the protective effect of an anti-RSV antibody. To assess the ability of these mAbs to interact with modern RSV strains circulating in St. Petersburg (Russia) between 2014 and 2022, 73 RSV-A and 22 RSV-B isolates were analyzed. Six mAbs were directed to conserved epitopes of the F protein as they interacted most efficiently with both RSV subtypes in a fixed cell-ELISA and could be used for diagnostic assays detecting RSV.

## 1. Introduction

Human respiratory syncytial virus (RSV) is a significant global health concern, particularly in young children, where it is the leading cause of severe respiratory illness [1,2,3]. Incidence data from 132 developed countries indicate that annually RSV infection leads to over 3 million hospitalizations and approximately 60,000 deaths among children under 5 years of age [4]. While RSV infection is most commonly detected in children under 2 years old, reinfections can occur throughout life. Certain populations, such as the elderly and adults with immunopathology or chronic cardio-pulmonary diseases, are at higher risk for repeated episodes and severe courses of RSV infection [5,6]. One unique aspect of RSV pathogenesis its ability to induce immunopathology in patients with pre-existing conditions, such as imbalanced cytokine backgrounds, allergies, or concomitant disease. This can lead to bronchial hyperreactivity, obstruction, and pulmonary eosinophilia [7]. Notably, RSV-induced bronchiolitis during infancy, even in the absence of a predisposition to allergies, increases the risk of recurrent episodes of bronchial obstruction and the development of bronchial asthma later in life [8].

Monoclonal antibodies (mAbs) have proven to be effective tools for diagnosing viral infections. Specifically, RSV targeted mAbs are being used in rapid point-of-care diagnostic tests that can be administered at the bedside. These tests offer accurate and efficient detection of RSV infections, enabling timely treatment and management [9,10], and mAbs can also be applied in clinical practice as a therapeutic drug [11]. In addition to its practical applications in medicine, virus-specific mAbs are widely used for research purposes, including the study of RSV pathogenesis. Researchers apply mAbs in immunohistochemical methods to characterize changes in the localization and activity of RSV proteins expressed in infected cells and tissues following exposure to the virus or various factors [12]. mAbs are also used to assess the reduction in virus replication and spread in cell culture when testing antiviral drugs in vitro [13].

The development of NGS technologies has led to a significant increase in interest in molecular epidemiology of RSV infection. It was established that the genetic and antigenic variability of RSV is influenced by various environmental factors, including positive selection of virus variants under pressure from the host’s immune system [14,15,16]. RSV exists as a single serotype but is divided into two antigenic subgroups (RSV-A and RSV-B) depending on the antigenic properties of the attachment protein G, as determined by ELISA using RSV-specific mAbs [17]. RSV strains within each subgroup exhibit pronounced genetic diversity. Human RSV-A is further classified into 9–12 genotypes, while up to 32 genotypes have been identified among RSV-B [18,19].

The envelope of the RSV virion includes three transmembrane glycoproteins: the attachment protein G, the fusion protein F, and the small hydrophobic protein SH. Virions bud from the cell surface as filamentous structures. The G-protein attaches to the cell, while the F protein facilitates fusion of the viral envelope with the cell membrane of neighboring infected and uninfected cells. Both F and G proteins play crucial roles in the induction of RSV-neutralizing antibodies [20].

Mutations in RSV surface glycoproteins can change the antigenic properties of the virus, allowing it to avoid neutralization, which contributes to reinfections and a complicated disease course [15,21,22]. Therefore, identification of the most immunogenic and evolutionary stable antigenic determinants in the structure of RSV proteins, that can induce a protective response to the ever-changing RSV variants, is crucial for the development of effective vaccines. Targeted site-specific mAbs are essential for studying the antigenic variability of RSV circulating in a population [23,24,25] and evaluating the functional role of individual determinants of RSV proteins [26,27]. They can also be used as passive immunization to protect from RSV.

Despite the recognized importance of RSV in infectious respiratory disease, there are very few drugs proven to be safe and effective for treatment of RSV infection. Their use is often limited by high toxicity and undesirable side effects. In this context, the development of virus-neutralizing mAbs is a promising approach for the prevention and treatment of RSV infection. Until very recently, the only drug used prophylactically in clinical practice to prevent severe RSV infection in at-risk children was palivizumab (PVZ) (Sinagis), which is a humanized RSV-neutralizing mAb, targeting the RSV F protein [11]. In 2023, the new recombinant human IgG1 kappa monoclonal antibody nirsevimab that interacts with conserved epitopes of the F1 and F2 subunits of RSV F protein proved its efficacy in preventing RSV [28]. This drug has been approved by the FDA for the prevention of RSV lower respiratory tract disease in neonates and infants born during or entering their first RSV season, and in children up to 24 months of age who remain vulnerable to severe RSV disease through their second RSV season [29].

Since the F glycoprotein exhibits relatively high antigenic stability, the development of new anti-RSV immunodrugs is focused on creating broad-spectrum mAbs that interact with this viral protein [30]. Previously, a panel of seven murine mAbs specific to RSV was developed at the Smorodintsev Research Institute of Influenza. These mAbs, named 9C5, RS-25, 4F2, 5F3, 5H8, 1H3, and 5F8, were shown to interact with the F protein in Western blotting [31,32]. Nine new RSV-specific murine mAbs have now been obtained: 7B12, 11G4, 12C8, 12C9, 9E12, 10G6, 7H8, 7H11, and 9H1. The aim of the present study was to characterize in depth the new panel of murine mAbs specific for the RSV F protein.

## 2. Materials and Methods

*Viruses*. Three reference RSV prototype strains belonging to different antigenic groups were selected for the present study: Long (RSV-A, isolated in 1956), provided by the National Institute for Medical Research (London), A2 (RSV-A, isolated in 1961) and 9320 (RSV-B, isolated 1977) were obtained from the Influenza Reagent Resource (IRR) collection (Department of Human Retrovirology, Academic Medical Center, University of Amsterdam, The Netherlands).

*Isolation of RSV from clinical samples* was performed in MA-104 cell culture (Russian collection of cell cultures of the Research Institute of Cytology of the Russian Academy of Sciences, St. Petersburg, Russia) using PCR-positive nasopharyngeal swabs. These samples were collected in infectious children’s hospitals in St. Petersburg, Russia in 2014–2022 from hospitalized newborns, infants, and children in the first two days after admission. The Local Ethic Committee approved investigation. All patients or their legal representatives signed an informed consent to participate in the study.

*Cultivation, purification, and concentration of RSV*. RSV strains were further cultivated in continuous cell culture MA-104 for 5–7 days in serum-free alpha-MEM medium (LLC BioloT, St. Petersburg, Russia). The virus-containing culture fluid was used to purify and concentrate the virus by ultracentrifugation in a sucrose density gradient. The protein content in the purified concentrate of RSV (RSVp) was determined using the Pierce^®^ BCA Protein assay kit (Thermo Scientific, Waltham, MA, USA).

*Obtaining mAbs specific to RSV*. The panel of RSV mAbs was obtained by immunization of BALB/c mice with RSVp, strain Long. Detailed methods of obtaining RSVp and mAbs were described previously [32]. The studies were carried out in strict accordance with the Directive for the Protection of Animals Used for Scientific Purposes [33].

*SDS electrophoresis of RSV proteins* was performed according to the Laemmli method [34] in a 5–15% polyacrylamide gradient after RSVp treatment under various conditions. Non-reducing conditions included heat treatment of RSVp in buffer (0.06 M Tris-HCl, pH 6.8; 2% SDS) for 2 min at 100 °C, and also for 20 or 90 min at 37 °C. Reducing conditions included incubation of the RSVp for 2 min at 100 °C in the same buffer with the addition of 5% β-mercaptoethanol (β-ME).

*Immunoblotting*. Following electrophoresis, viral proteins were transferred from the gel onto a nitrocellulose membrane (NM) (0.45 µm, Bio-Rad, Munich, Germany) in transfer buffer (20 mM Tris, 192 mM glycine, 20% ethanol, pH 8.3) at 100 V for 1 h. Free points on the membrane were blocked at 4 °C for 18 h with 5% skimmed milk (IP Hiba M.A., Moscow, Russia), diluted with 0.01 M phosphate-buffered saline (PBS), pH 7.2, with the addition of 0.05% Tween-20 (blocking solution FSB-M). Incubation of mAbs (at a concentration of 5–10 μg/mL, diluted with PBS-M) with viral proteins on NM was carried out at 3 7 °C for 2 h. F-specific humanized mAb PVZ (Behringer Iingelheim Pharma GmbH & Co. KG, Ingelheim, Germany) was used as the reference drug. After that NM-strips were treated at 37 °C for 2 h with peroxidase conjugated goat anti-mouse IgG (gam-Px) (Sigma, Livonia, MI, USA) for characterized mAbs or with peroxidase conjugated human IgG specific mAb 5A9 (5A9-Px) (Polygnost LLC, St. Petersburg, Russia) for PVZ imaging. The visualization of Px on NM was performed with a substrate solution containing 3,3′,5,5′-tetramethylbenzidine (TMB) (Life Technologies, Waltham, MA, USA). The molecular weights (MW) of proteins were estimated from a calibration curve using electrophoretic markers (Sigma color burst C1992, Sigma, USA).

*Cell-ELISA* was used to assess the effectiveness of the interaction of mAbs with intracellular RSV. Monolayers of MA-104 cells grown in 96-well culture plates (Jet Bio-Filtration Co., Ltd., Guangzhou, China) were infected with RSV at a dose of 100 TID_50_. After 5–7 days of cultivation at 37 °C in a CO_2_ incubator (Sanyo, Tokyo, Japan) and after the development of a pronounced cytopathic effect, the cells were fixed with 80% chilled acetone for 20 min. mAbs diluted with 5% PBS-M was added to the wells with fixed cells and incubated for 2 h at 37 °C. Virus-bound mAbs were detected by adding gam-Px. 5A9-Px was used to visualize PVZ. The Px reaction was visualized by adding a substrate mixture containing 0.1 mg/mL TMB (Sigma, USA) and 0.02% H_2_O_2_ in acetate–citrate buffer, pH 5.0 (AO LenReaktiv, St. Petersburg, Russia). The optical density was measured after stopping the reaction with 2N H_2_SO_4_ on a Multiskan MS photometer (Labsystems, Vantaa, Finland) at a wavelength of 450 nm (OD_450_).

*The evaluation of the virus-neutralizing activity of mAb in monolayer MA-104 culture* was described earlier [32]. Inhibition of RSV replication in the presence of mAbs was measured by cell-ELISA using the mAb 9C5-Px conjugate (1/2000 in PSB-M) prepared in house. The final neutralizing titer was determined by the lowest concentration of mAbs that caused two-fold decrease in OD_450_ values compared to the control of virus reproduction (infected cells without the addition of mAbs).

The conjugates of the mAbs with horseradish peroxidase (HRP) were prepared by the periodate method [35].

*Competitive ELISA*. ELISA plates (Medpolimer, St. Petersburg, Russia) were sensitized with RSVp (5 µg/mL). Various combinations of mAbs conjugated with HRP (mAbs-Px) and unlabeled mAbs (ten-fold dilutions in PBS-M) were mixed in equal volumes (50 μL) and transferred to the wells. The plates were incubated for 18 h at 4 °C. The degree of inhibition of mAb-Px binding to RSV in the presence of unlabeled mAb was determined by the following formula:

$$\left[1-\left(\frac{{\mathrm{OD}}_{450\;}\mathrm{test}-{\mathrm{OD}}_{450\;}K^{-}}{{\mathrm{OD}}_{450\;}K^{+}-{\mathrm{OD}}_{450\;}K^{-}}\right)\right]\times 100\;\left(\%\right)$$

OD_450_ test represents the OD_450_ of the analyzed sample when a mixture of mAbs-Px/unlabeled mAb was added to RSV;

OD_450_ K^−^ (negative control) represents the OD_450_ in wells without added virus;

OD_450_ K^+^ (100% virus/mAb interaction, positive control) is OD_450_ obtained in wells with RSV by adding mAbs-Px in the absence of unlabeled mAbs.

*Determination of the mAbs isotype* was carried out in indirect ELISA. RSVp Long strain (5 μg/mL) was absorbed on the plates with the following addition of RSV-specific mAbs. mAbs bound to the virus were detected by sequential addition of goat anti-idiotypic serum against murine IgG and Px-conjugated rabbit anti-goat IgG (a set of reagents Sigma-Aldrich, Burlington, MA, USA)

*Selection of RSV escape mutants (EMs).* RSV strain Long (1–10 TID_50_) was cultivated in the presence of a high concentration (2–5 mg/mL) of neutralizing RSV-specific mAbs in the growth medium. mAbs/RSV mixtures were incubated for an hour at 37 °C, then they were added to plates with MA-104 cell culture (100 μL of the mixture per well). After 5–7 days of cultivation in a CO_2_ incubator at 37 °C, infected cells containing single symplasts were taken from the wells and used for infection of MA-104 cells for 3–4 times with adding of corresponding mAbs. The virus obtained after repeated cultivation in the presence of mAbs was sequenced to identify mutations that allow RSV to escape the interaction with neutralizing antibodies.

*Real-time PCR with reverse transcription (rtRT-PCR).* The virus-containing culture medium obtained by isolation of RSV from clinical samples (1–2 serial passages in MA-104 cell culture) was used for molecular detection of RSV. To identify specific fragments of RSV nucleic acids, the AmpliSens Reverta and Ampli-Sens^®^ ARVI-screen-FL reagent kits (InterLabService, Moscow, Russia) were used in accordance with the instructions of the manufacturer. rtRT-PCR detection was performed to identify the RSV-A and RSV-B subgroups using the QIAGEN One-step RT-PCR kit (QIAGEN, Hilden, Germany). Amplification of F- and G-genes was carried out using primers designed in-house. Amplification products were purified using the QIAquick PCR purification Kit (Qiagen, Germany). The molecular weight of DNA amplified fragments after gel electrophoresis in agarose was determined using a marker (1 kb, Thermo Scientific, USA).

*Sequencing of F- and G-genes of RSV isolates* was performed by the Sanger method using a commercial ABI PRISM BigDye Terminator v3.1 Cycle Sequencing Kit (Applied Biosystems, Foster City, CA, USA) on a GA 3130 genetic analyzer (Applied Biosystems, USA). Primers for sequencing were the same as for rtRT-PCR. The products of the sequencing reaction were purified using the BigDye XTerminator Purification Kit (Applied Biosystems, USA). Nucleotide sequences were determined using a 4-channel automated system for capillary electrophoresis and fluorescent detection of DNA fragments ABI prism 3130 Genetic Analyzer (Applied Biosystems, USA). Capillary electrophoresis was carried out in ABI prism 3130 POP-7 polymer (Applied Biosystems, USA). Nucleotide sequences were assembled using the Vector NTI 10 Advance program ContigExpress (Invitrogen, Carlsbad, CA, USA). Sequence alignment and phylogenetic analysis (building phylogenetic trees by the maximum likelihood method and the Tamura-3 model with gamma distribution, bootstrap 1000) were performed using the MEGA7 program.

*Statistical processing of results*. Comparison of sample proportions (detecting the frequency characteristic) was performed using Fisher’s exact test. The null hypotheses tested by the criteria were rejected at a significance level of *p* < 0.05.

## 3. Results

### 3.1. Interaction of the mAbs with Reference RSV Strains Using a Cell-ELISA

Sixteen mAbs were analyzed in this study. Seven of them—9C5, RS-25, 4F2, 5F3, 5H8, 1H3, and 5F8—were described previously while nine new RSV-specific mAbs—7B12, 11G4, 12C8, 12C9, 9E12, 10G6, 7H8, 7H11, and 9H1 were generated within this study. RSV (Long strain, subgroup A) was used as immunogen for obtaining all the mAbs. According to cell-ELISA 11 mAbs out of 16 showed intense interactions not only with RSV Long strain but also with other reference viruses, including the A2 (RSV-A) strain and the 9320 (RSV-B) strain. Some of these eleven mAbs could be target conserved subtype-independent regions of the RSV surface proteins. Two mAbs (5F8 and 5H8) exhibited weaker interaction with the reference viruses from both antigenic groups, including the Long strain. mAbs 5F3, 7H8, and 1H3 detected both RSV-A reference strains in cell culture but interacted with RSV-B strains weakly, indicating a subgroup-specific preference (Table 1).

The nine newly obtained mAbs were tested for cross-reactivity with heterologous viruses using a cell-ELISA in which cells were infected with influenza A, B, para-influenza viruses (2 and 3) and adenovirus. The results showed that none of these mAbs exhibited any cross-reactivity with these viruses. Additionally, the mAbs did not interact with cellular antigens.

In an indirect ELISA using RSVp Long strain as the capture antigen, all mAbs were found to belong to different IgG isotypes. Evaluation of the neutralizing activity revealed that three of the previously obtained mAbs (5F8, 5F3, and 5H8), as well as five new ones (7B12, 12C9, 9E12, 10G6, and 7H8), had virus neutralizing activity against RSV group A reference strains. They also showed some level of neutralizing activity against RSV-B, but to a reduced extent. However, none of the resulting mAbs was as potent as the commercial virus-neutralizing antibody PVZ (Table 2).

### 3.2. Relationship between Conformational Changes in the Structure of the F Protein and the Ability of mAbs to Interact with It

**Figure 1:**
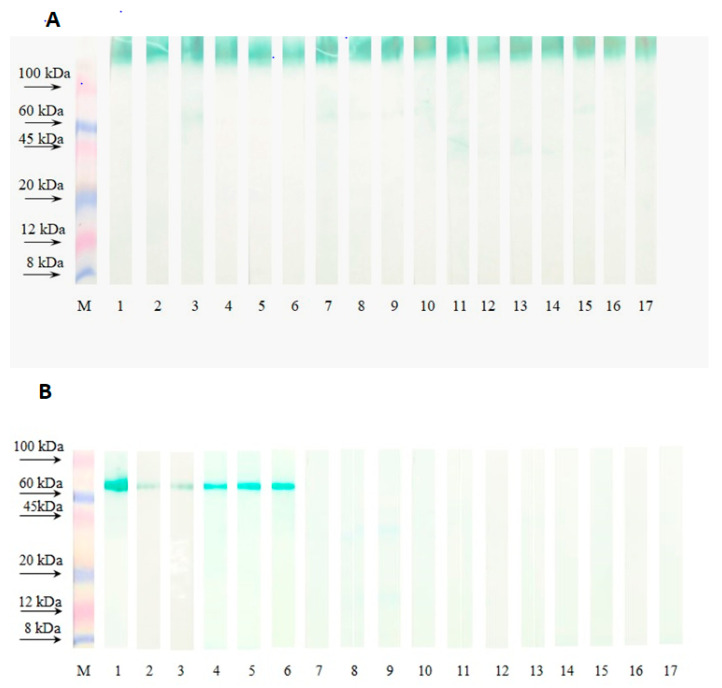
Interaction of mAbs with the F protein of the RSV Long strain after treatment of the purified RSV concentrate under various conditions (immunoblotting). (**A**)—mild heat treatment of the virus under non-reducing conditions (20 min at 37 °C without β-ME), (**B**)—boiling of the virus under non-reducing conditions (2 min at 100 °C without β-ME). mAbs concentration was 5 µg/mL. Tracks: mAbs RS-25 (1), 9C5 (2), PVZ (3), 11G4 (4), 10G6 (5), 9H1(6), 4F2 (7), 5F3 (8), 5H8 (9), 1H3 (10), 5F8 (11), 7B12 (12), 12C8 (13), 12C9 (14), 9E12 (15), 7H8 (16), 7H11 (17). M-markers (Sigma color burst C1992).

The relationship between mAbs binding to RSV and the conformation of the target protein was also assessed. After electrophoresis of RSVp treated under the mildest non-reducing conditions (for 20 min at 37 °C without disrupting disulfide S=S bonds), all mAbs interacted on the immunoblot equally well. The observed wide colored diffuse bands are typical for interaction of antibodies with highly glycosylated high-molecular protein structures. The MW of this band was determined as 140 to 190 kDa (Figure 1A), which is characteristic for the conformationally native oligomeric (homodimeric and homotrimeric) forms of the RSV F-glycoprotein [36,37].

With an increase in the time of mild heat treatment of RSVp under non-reducing conditions (37 °C for 90 min without β-ME), the oligomeric structure of F protein was partially destabilized to a dimeric form only (MW of 135–140 kDa) [36,38]. All of the mAbs also interacted on NM in blotting with this pattern, but with varying intensity. The mAbs RS25, 5H8, 7H8, and 9H1 showed the highest binding activity (Table 2).

When the RSVp was boiled for a short period (2 min) under non-reducing conditions, only five out of the 16 mAbs interacted with a protein band with a MW of 65–70 kDa. Among these mAbs, only one (10G6, track 5) exhibited neutralizing activity (Figure 1B). This MW corresponds to an uncleaved precursor of the F protein (F0) or a cleaved F0 molecule with preserved disulfide bonds between the F1 and F2 subunits, existing in a monomeric form.

None of the mAbs, including PVZ (lane 3), reacted with the primary amino acid sequence of the F protein, which lost its spatial organization as a result of RSV treatment in severe reducing conditions, boiling in the presence of β-ME (Table 2).

Thus, all mAbs showed dependence on the conformational structure of their target epitopes within the F protein molecule. At the same time, this characteristic was particularly evident in the neutralizing mAbs (5F3, 5H8, 5F8, 7B12, 12C9, 9E12, 10G6, 7H8).

### 3.3. Determination of Epitope Targeting of mAbs

To characterize the epitope targeting of the obtained mAbs a competitive ELISA was performed. For this purpose, conjugates of mAbs with horseradish peroxidase (mAbs-Px) were prepared. The degree of inhibition of virus binding by mAbs-Px was assessed in the presence of homologous or heterologous “unlabeled” mAbs. The degree of mAbs-Px binding to RSV in the absence of the unlabeled competing mAbs was considered as 100% (positive control, OD_450_ K+). The degree of mAbs-Px binding to RSV when adding an equivalent amount of the same unlabeled mAbs at a working concentration (0.05–0.1 μg/mL) varied between 60 and 94% depending on the mAbs analyzed.

To assess the threshold for non-specific inhibition of F-specific antibodies, the mAb 8B10 targeting the N-protein of RSV was used. mAb 8B10 was obtained and characterized earlier at the Smorodintsev Research Institute of Influenza. The non-specific inhibition ranged from 7% to 26%. In accordance with this an inhibition index of more than 40% of K+ indicated the presence of common antigenic determinants (1.5 times the maximum possible level of nonspecific interaction).

Evaluation of competitive interactions revealed that commercial PVZ and mAbs 5H8, 1H3 and 5F8 did not have a common binding site with each other or with other mAbs. The other mAbs demonstrated partial overlap of their recognition sites. The non-neutralizing mAbs 9C5 and RS25 significantly (more than 80%) inhibited each other, as well as the non-neutralizing mAbs 4F2, 7H11, 11G4, and 12C8 with varying intensity. mAb 4F2 competed with 9C5, RS25, and 7H11. mAb 11G4 had cross-interactions with non-neutralizing mAbs 12C8 and 9H1 in addition to binding to epitopes recognized also by 9C5 and RS25. The mAb 9H1 significantly inhibited binding of mAb 11G4 only. Neutralizing mAb 7B12 shared binding sites with neutralizing mAbs 12C9 and 10G6, which did not compete with each other. Neutralizing mAb 9E12 had common determinants with neutralizing mAbs 5F3 and 7H8. Interestingly, some of mAbs exhibited cross-interactions with both neutralizing and non-neutralizing mAbs. For example, mAbs 7H11 and 12C8 competed not only with each other and non-neutralizing mAbs 9C5, RS25, 4F2 (only mAbs 7H11), 11G4 (only mAbs 12C8) but also with the neutralizing mAbs 5F3 and 7H8. Identical properties were shown for 5F3 and 7H8, as they competed with each other, as well as with the neutralizing mAb 9E12 and non-neutralizing mAbs 7H11 and 12C8.

**Figure 2:**
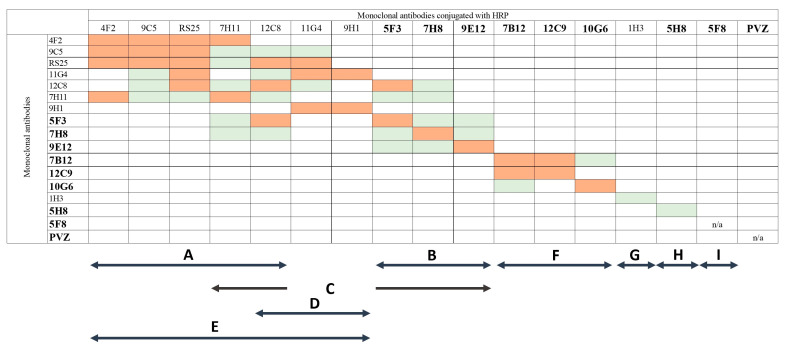
Competitive interaction between mAbs specific to RSV F protein (cell-ELISA). Neutralizing mAbs are highlighted in bold; n/a—were not analyzed due to inability to obtain mAbs-Px. The arrows under the figure (A–I) indicate the putative antigenic sites. Colors in the table indicate the rate of inhibition of mAbs-Px binding to RSV in the presence of unlabeled mAb: white—less than 40% (lack of competition), green—41 to 60%, orange—61 to 95% inhibition.

The detailed results of the competitive interactions are summarized schematically in Figure 2.

**Figure 3:**
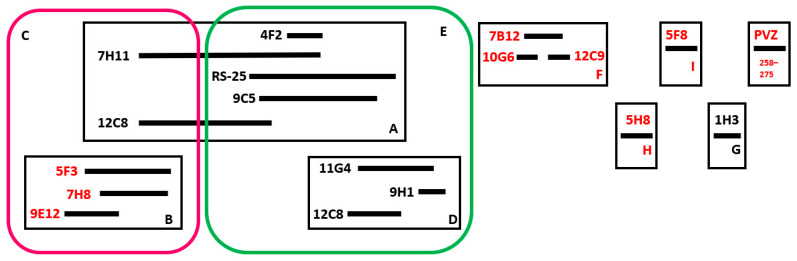
Proposed arrangement of target epitopes of mAbs in F protein of RSV. The neutralizing mAbs and antigenic determinants are highlighted in red.

Based on the data obtained for mAbs competition with each other, as well as data on their neutralizing activity, a graphical scheme of the probable relative arrangement of RSV F protein sites was proposed (Figure 3). Nine sites of interaction on the RSV F protein were identified. Of these, four turned out to be isolated: three neutralizing sites F, H, and I and one non-neutralizing site G. Five partially overlapping sites (A, B, C, D, E) may represent one antigenic determinant with a complex spatial organization, including both neutralizing and non-neutralizing epitopes. The neutralizing site B is part of the C site, which also includes a part of the non-neutralizing target epitopes for mAbs 7H11 and 12C8. Epitopes A, D, E are targets for non-neutralizing mAbs. At the same time, none of the mAbs characterized in this study competed with the PVZ for binding to the F protein. It should be noted that, based on the results of mAbs competition, the 12C8 non-neutralizing epitope turned out to be structurally discrete, including two sequences distant from each other in sites A and D (Figure 3).

### 3.4. Analysis of Antigenic Variability of Currently Circulating RSV

The reference strains of RSV-A (Long, A2) and RSV-B (9320), which were used to evaluate the properties of the resulting mAbs, were isolated in the 1950–1970s. RSV of both antigenic subgroups, which circulated in Russia in recent decades, had diverged significantly from their progenitors and belonged to other genetic groups. RSV surface glycoproteins, including F, have undergone substantial changes [39,40]. These changes could potentially impact the interaction between modern viruses and mAbs derived from their evolutionary predecessors.

To investigate this assumption, the binding efficiency of mAbs to RSV isolates was assessed using cell-ELISA. A total of 73 RSV-A and 32 RSV-B strains, isolated from clinical materials (nasopharyngeal swabs) obtained from children hospitalized in clinics in St. Petersburg during 2014–2022, were included in the analysis. All isolates were previously typed as RSV-A or RSV-B by real-time RT-PCR.

The results are presented in Table 3. It was shown that the proportion of isolated RSV strains with decreased interaction with each of the F-specific mAbs (by at least 30%), compared to the positive control (K+). To standardize the presentation of results the OD_450_ values detected in the cell-ELISA during the interaction of each mAb with the reference strains (Long for RSV-A or 9320 for RSV-B) were taken as 100% (K+). A decrease in the interaction indicated the presence of antigenic changes in the target epitopes of modern isolates compared to their evolutionary predecessors, and also reflected the variability of epitopes within each RSV antigenic group.

Analysis of the results showed that six mAbs (RS25, 4F2, 7H11, 12C8, 11G4, and 9H1) interacted with high activity in cell-ELISA not only with the reference viruses but also with almost all “wild” isolates of both RSV-A and RSV-B. These mAbs determined evolutionary, highly conserved group-independent antigenic determinants (or one conformationally complex antigenic determinant) of F protein similar to those observed for PVZ. It is important to note that all six of these mAbs are non-neutralizing.

The epitopes for binding neutralizing mAbs 9E12 (part of site B) and 5H8 (site H) showed equal variability for RSV-A and RSV-B isolates. Changes in the target epitopes for mAbs 9E12 and 5H8 were observed, respectively, in 24–34% and 58–68% of strains isolated in 2014–2022.

In current RSV-A strains, three sites on the F protein (F, I, and part of B for mAbs 5F3 and 7H8) that interact with neutralizing mAbs exhibited a decrease in binding activity to 42–80% of isolates, indicating a fairly high level of variability. However, within the RSV-B group, these sites showed antigenic stability with variability observed in only 18–25% of cases. Additionally, the target epitope G for non-neutralizing mAb 1H3 was also stable within the RSV-B group.

The mAbs RS25 and 9C5 provide an interesting example of the influence of target epitope conformation on mAb properties. Competitive ELISA showed that the target antigens for these two mAbs shared more than 85% structural similarity. They also competed for binding with the same mAbs (Figure 3). However, immunoblotting results indicated that mAb 9C5 exhibited greater conformational dependence compared to RS25, as it reacted weaker with conformationally changed forms of the F protein (Table 2). These differences may explain why mAb 9C5 unlike mAb RS25 weakly reacted with 18–32% of RSV isolates (Table 3), despite having a largely identical target on the F protein molecule.

For one of the neutralizing independent epitopes 5F8, it was possible to identify a critical position in the structure of the F protein, which is necessary for its effective binding to specific mAb. By selection, as a result of virus cultivation in the presence of a high content of neutralizing mAbs in the growth medium, an escape mutant (EM) of the virus—immunogen (Long strain)—was obtained, which is insensitive to the neutralizing effect of mAb 5F8. Sequencing of the RSV RNA coding the F protein showed that, compared with the original strain, EM had one substitution, N240S, indicating that N240 was essential for binding.

## 4. Discussion

This study characterized a panel of 16 mAbs that specifically interact with RSV F protein, eight of which had virus-neutralizing activity.

RSV strains with identical primary structures of the F protein can exhibit different interactions with F-specific mAbs depending on the conformation of the binding target [41]. Our data also showed differences in the activity of mAbs RS25 and 9C5, which target almost the same epitope, possibly with a slight shift in the site of interaction. However, mAb 9C5 appeared to be more dependent on the spatial organization of the F protein. This may explain the lower efficiency of the interaction between mAb 9C5 and RSV isolates from both antigenic groups compared to mAb RS25. It is possible that mAb 9C5 binds to a labile part of the epitope, which undergoes conformational changes in modern circulating viruses.

Based on the results of competitive ELISA and neutralization assays, we proposed a hypothetical arrangement of mAbs target sites on the RSV F protein. We identified five partially overlapping sites, which may represent a complex spatial structure of one antigenic determinant, including one neutralizing and four non-neutralizing epitopes. Four sites (three neutralizing and one non-neutralizing) were found to be distinct. Importantly, none of the mAbs that we obtained competed for binding with neutralizing mAb PVZ, which targets an antigenic site on the F protein (amino acid sequence 262–276) [42,43].

Through competition assays, we discovered that the binding site for the non-neutralizing mAb 12C8 is structurally discrete and spatially divided into two distant sequences, likely due to the complex conformation of the F protein. The discreteness of antigenic determinants is not unique to RSV. For example, the Ø (zero) site includes fragments of two subunits of the F protein: F1 (sequence 196–231) and F2 (sequence 62–69) [21,44].

Six non-neutralizing mAbs (RS25, 4F2, 7H11, 12C8, 11G4, and 9H1) interacted in the cell-ELISA with high efficiency, slightly inferior to that detected for PVZ, with almost all isolates of 2014–2022, both RSV-A and RSV-B. These mAbs determined evolutionarily, highly conserved group-independent antigenic determinants of the F protein, making them candidates for use in various RSV detection methods and in tests for identification of RSV. On the contrary, four sites to which the neutralizing mAbs 5F3/7H8, 7B12/12C9/10G6, and 5F8, as well as the non-neutralizing mAb 1H3, are directed, were characterized by variability, with significantly higher degrees of variability observed in RSV-A compared to RSV-B.

These data indicate a greater variability in modern circulating RSV-A compared to RSV-B. Furthermore, the antigenic determinants that induce the synthesis of neutralizing antibodies are variable. One method for identifying sequences of RSV proteins that induce the synthesis of antiviral antibodies is the sequencing of escape mutants that are resistant to the action of neutralizing mAbs [23,25,45].

We obtained an RSV-A escape mutant (Long strain) through selection in the presence of mAb 5F8, in which an amino acid substitution N240S was detected in the F protein. Position 240 in the primary sequence of the F protein is located between the known antigenic sites Ø (zero) (amino acid sequence 196–231) and II (amino acid sequence 255–278) [21,43,45]. The amino acid residue at position 240 is not part of the six antigenic sites of the RSV F protein described in the literature that induce the synthesis of virus-specific antibodies. In one study, the F sequence of the 207–254 protein, which includes N240, was positioned as an epitope with an unknown function [46]. On the other hand, the nearby epitope 222–237 was identified as neutralizing [47]. Additionally, in 2016, a new antigenic site with sequence 178–240 was identified that induces antibody synthesis during primary infection in children [48]. It is possible that the epitope of interaction with neutralizing mAb 5F8, with the amino acid residue at position 240 critical for binding, belongs to this site. It is also possible that it forms an independent neutralizing site.

Given the high prevalence of RSV infection in Russia [3,49], there is a paramount need to create effective non-toxic drugs with specific anti-RSV activity, as well as sensitive diagnostic tests.

The protective properties of emerging antibodies in the body of an infected host depend on the structure of the viral antigens that induce its synthesis. Thus, the study of RSV evolution and virus variability is also of practical importance. Antigenic changes in RSV should be taken into account when testing vaccines under development. However, the antigenic variability of RSV circulating in different regions of Russia has not been extensively studied.

The characterization of the genetic variability of viruses is successfully solved by sequencing methods, which are currently widely used in laboratory practice. However, the antigenic properties of RSV can only be assessed using virus-specific mAbs [23,24,45,50], but their use is limited due to their high commercial price. Additionally, the spectrum of commercial RSV-specific mAbs with identified binding sites is also insufficient for full-fledged research.

Therefore, the creation of a broad panel of characterized and available RSV-specific mAbs could make a significant contribution to solving these issues.

## Figures and Tables

**Table 1:** Interaction of mAbs with RSV reference strains (cell-ELISA).

**RSV Strain**	**Subtype**	**OD_450_ for mAbs:**																
		**5F3**	**7H8**	**9E12**	12C8	4F2	7H11	RS25	9C5	11G4	9H1	**10G6**	**7B12**	**12C9**	**5F8**	**5H8**	1H3	**PVZ**
**Long**	RSV-A	0.89 ± 0.08	0.91 ± 0.08	1.10 ± 0.09	0.87 ± 0.09	0.90 ± 0.02	0.98 ± 0.10	0.98 ± 0.07	0.95 ± 0.09	0.84 ± 0.07	0.98 ± 0.05	0.90 ± 0.09	0.73 ± 0.03	0.95 ± 0.10	0.58 ± 0.02	0.59 ± 0.02	0.95 ± 0.02	1.11 ± 0.11
**A2**	RSV-A	0.80 ± 0.06	0.72 ± 0.07	0.75 ± 0.03	0.77 ± 0.05	0.87 ± 0.03	0.70 ± 0.07	0.92 ± 0.05	0.84 ± 0.07	0.73 ± 0.04	0.79 ± 0.06	0.71 ± 0.03	0.75 ± 0.05	0.71 ± 0.03	0.53 ± 0.06	0.56 ± 0.04	0.75 ± 0.02	1.25 ± 0.09
**9320**	RSV-B	0.49 ± 0.02	0.48 ± 0.04	0.72 ± 0.04	0.74 ± 0.04	0.89 ± 0.07	0.74 ± 0.04	0.90 ± 0.08	0.76 ± 0.06	0.86 ± 0.06	0.81 ± 0.07	0.76 ± 0.05	0.78 ± 0.03	0.80 ± 0.03	0.51 ± 0.02	0.43 ± 0.03	0.51 ± 0.03	1.14 ± 0.08

Note: The cell-ELISA represents the mean OD_450_ ± SD of three measurements for each mAb at a final concentration of 5 µg/mL when cell cultures were infected with virus in a dose of 100 TCID_50_ per well. Neutralizing mAbs are indicated in bold. OD_450_ obtained for mAbs with reduced activity are highlighted in gray (decrease in OD_450_ by at least 40% relative to the highly active mAb RS25).

**Table 2:** Characteristics of mAbs obtained against the RSV.

mAb	mAb Isotype	Neutralizing mAb Activity (μg/mL) upon Interaction with RSV in Cell Culture (Cell-ELISA Data):			Color Intensity of F Protein Interaction with mAbs on Nitrocellulose Membranes after the Following Treatment:			
					Non-Reducing Treatment			Reducing Treatment
		RSV-A Long	RSV-A A2	RSV-B 9320	Heat for 20 min, 37 °C w/o β-ME ^1^	Heat for 90 min, 37 °C w/o β-ME ^2^	Boiling for 2 min, 100 °C, w/o β-ME ^3^	Boiling for 2 min, 100 °C, with β-ME ^4^
9C5	IgG2a	˃800	˃800	˃800	++++	++	+	−
RS25	IgG2b	˃650	˃650	˃650	++++	+++	+++	−
4F2	IgG1	˃700	˃700	˃700	++++	+	−	−
5F3	IgG1	16	26	74	++++	+	−	−
5H8	IgG1	135	135	270	++++	+++	−	−
1H3	IgG1	˃900	˃900	˃900	++++	+	−	−
5F8	IgG2a	9	13	54	++++	++	−	−
7B12	IgG2a	63	63	126	++++	++	−	−
11G4	IgG1	˃650	˃650	˃650	++++	++	++	−
12C8	IgG2b	˃650	˃650	˃650	++++	+	−	−
12C9	IgG1	56	56	56	++++	+	−	−
9E12	IgG1	63	63	252	++++	+	−	−
10G6	IgG1	125	125	250	++++	++	++	−
7H8	IgG1	75	75	300	++++	+++	−	−
7H11	IgG2a	˃800	˃800	˃800	++++	+	−	−
9H1	IgG1	˃550	˃550	˃550	++++	+++	++	−
PVZ	IgG1	0.1	0.1	0.2	++++	++	+	−

Note: RSV-neutralizing mAbs are highlighted in gray. The color intensity of the F protein replica on NM as a result of interaction with mAbs was assessed using the 4-cross system: (+) weak interaction, (++) moderate interaction, (+++) intense interaction, (++++) very strong interaction, (−) lack of interaction with the mAb. RSVp Long strain was used as the antigen for electrophoresis. w/o—without F protein form for mAbs binding: ^1^ trimer (MW 140–190 kDa) ^2^ dimer (MW 135–140 kDa) ^3^ monomer (MW 65–70 kDa) ^4^ monomer divided to two subunits (MW 50 kDa and 20 kDa).

**Table 3:** Variability of antigenic determinants of the F protein of RSV strains isolated in seasons 2014–2022.

**Antigenic Group**	**Number of Strains**	Proportion of Strains with Reduced Interaction with F-Specific mAbs (%) *:																
		4F2	9C5	RS25	7H11	12C8	11G4	9H1	**5F3**	**7H8**	**9E12**	**7B12**	**12C9**	**10G6**	**5H8**	**5F8**	1H3	**PVZ**
RSV-A	73	0	32.8	0	0	1.4	1.4	0	**42.5**	**52.1**	24.7	**79.5**	**80.8**	**72.6**	58.9	**75.3**	**41.1**	0
RSV-B	32	0	18.8	0	0	3.1	0	0	18.8	18.8	34.4	3.1	18.8	12.5	68.8	25.0	15.6	0
Level of significance of differences (*p*) between the RSV-A and RSV-B groups		*p* ˃ 0.05	*p* ˃ 0.05	*p* ˃ 0.05	*p* ˃ 0.05	*p* ˃ 0.05	*p* ˃ 0.05	*p* ˃ 0.05	*p* < 0.001	*p* < 0.001	*p* ˃ 0.05	*p* < 0.0001	*p* < 0.0001	*p* < 0.0001	*p* ˃ 0.05	*p* < 0.0001	*p* < 0.01	*p* ˃ 0.05

* Reduction in OD_450_ values in cell-ELISA during the interaction of mAbs with RSV-A or RSV-B isolates by at least 30% compared to the reference strains Long or 9320, respectively. Neutralizing mAbs are highlighted in bold in the title. Group-specific antigenic determinants are highlighted in different colors: mAbs 5F3 and 7H8 interacted with the putative antigenic site B (yellow); mAbs 7B12, 12C9, and 10G6—with antigenic site F (green); mAbs 5F8 and 1H3—with sites I (pink) and G (blue), respectively.

## Data Availability

Data on obtained monoclonal antibodies is described in details in ref. [31,32]. Other data sharing is not applicable to this article.
